# Galectin-3 Deficiency Facilitates TNF-α-Dependent Hepatocyte Death and Liver Inflammation in MCMV Infection

**DOI:** 10.3389/fmicb.2019.00185

**Published:** 2019-02-08

**Authors:** Bojana Stojanovic, Jelena Milovanovic, Aleksandar Arsenijevic, Bojan Stojanovic, Ivana Strazic Geljic, Nebojsa Arsenijevic, Stipan Jonjic, Miodrag L. Lukic, Marija Milovanovic

**Affiliations:** ^1^Center for Molecular Medicine and Stem Cell Research, Faculty of Medical Sciences, University of Kragujevac, Kragujevac, Serbia; ^2^Faculty of Medical Sciences, Institute of Pathophysiology, University of Kragujevac, Kragujevac, Serbia; ^3^Faculty of Medical Sciences, Institute of Histology, University of Kragujevac, Kragujevac, Serbia; ^4^Department of Surgery, Faculty of Medical Sciences, University of Kragujevac, Kragujevac, Serbia; ^5^Department for Histology and Embryology, Center for Proteomics, Faculty of Medicine, University of Rijeka, Rijeka, Croatia

**Keywords:** galectin-3, hepatitis, murine cytomegalovirus infection, TNF-α, hepatocyte death

## Abstract

Galectin-3 (Gal-3) has a role in multiple inflammatory pathways. Various, opposite roles of Gal-3 in liver diseases have been described but there are no data about the role of Gal-3 in development of hepatitis induced with cytomegalovirus infection. In this study we aimed to clarify the role of Gal-3 in murine cytomegalovirus (MCMV)-induced hepatitis by using Gal-3–deficient (Gal-3 KO) mice. Here we provide the evidence that Gal-3 has the protective role in MCMV-induced hepatitis. Enhanced hepatitis manifested by more inflammatory and necrotic foci and serum level of ALT, enhanced apoptosis and necroptosis of hepatocytes and enhanced viral replication were detected in MCMV-infected Gal-3 deficient mice. NK cells does not contribute to more severe liver damage in MCMV-infected Gal-3 KO mice. Enhanced expression of TNF-α in the hepatocytes of Gal-3 KO mice after MCMV infection, abrogated hepatocyte death, and attenuated inflammation in the livers of Gal-3 KO mice after TNF-α blockade suggest that TNF-α plays the role in enhanced disease in Gal-3 deficient animals. Treatment with recombinant Gal-3 reduces inflammation and especially necrosis of hepatocytes in the livers of MCMV-infected Gal-3 KO mice. Our data highlight the protective role of Gal-3 in MCMV-induced hepatitis by attenuation of TNF-α-mediated death of hepatocytes.

## Introduction

Infection with human cytomegalovirus (HCMV) is typically asymptomatic in immunocompetent individuals, but in individuals with immature or deficient immune system HCMV is a cause of morbidity and mortality (Mocarski et al., 2007). In immunocompromised hosts, especially in transplant recipients, HCMV induces severe hepatitis that elevates mortality (Bozza et al., 2007; Mocarski et al., 2013). A better understanding of HCMV-induced liver damage could provide insights into potential novel therapeutic strategies for immunocompromised patients (Livingston-Rosanoff et al., 2012). Murine CMV (MCMV) causes a chronic infection with initial hepatic inflammation and damage and is the most widely used model to study the pathogenesis of human CMV disease (Livingston-Rosanoff et al., 2012; Brune, 2013). In C57BL/6 mice NK cells, recruited by inflammatory monocytes, play a crucial role in the early control of MCMV infection by both NK cell-mediated cytotoxicity and the production of effector cytokines (Smith et al., 2002; Scalzo and Yokoyama, 2008). Infection promotes liver inflammation by enhancing IL-1β and TNF-α production. TNF-α plays a role in defense against MCMV infection by activation of antiviral activities of T and NK cells (Orange and Biron, 1996), but is also critical in MCMV-induced liver damage in normal or immunodeficient hosts (Orange et al., 1997). TNF-α-mediated signaling is required for the development of early necrotic foci in the livers of MCMV-infected C57BL/6J Rag-/- mice and the NK- and T- cell-deficient E26 mice, indicating that the main source of TNF-α are non-immune liver cells.

Galectins play an important role in the regulation of major cellular functions such as cell attachment, spreading, migration, proliferation (Elola et al., 2007). Gal-3 also regulates cell signaling and apoptosis (Markowska et al., 2011), and plays different roles in the pathogenesis of many inflammatory, infectious and malignant diseases (Radosavljevic et al., 2011; Volarevic et al., 2012; Arsenijevic et al., 2016; Simovic Markovic et al., 2016). An increased expression of Gal-3 was observed in human T lymphotropic virus-1 infection (Hsu et al., 1996), as well as in Junin virus-induced central nervous system lesions, but its role in the pathogenesis of viral disease is not revealed (Jaquenod De Giusti et al., 2011).

We have previously shown that deletion of galectin-3 gene, Lgals3, prevents ConA-induced hepatitis and that Gal-3 regulates the capacity of dendritic cells to promote NKT cell induced liver injury (Volarevic et al., 2012, 2015). Further, Lgals3 ablation enhances liver steatosis, but attenuates inflammation and IL-33 dependant fibrosis in mouse model of non-alcoholic fatty liver disease (NAFLD) (Jeftic et al., 2015), and enhances bile duct damage and liver fibrosis in xenobiotic induced primary biliary cholangitis (PBC) (Arsenijevic et al., 2016). Also Gal-3 overexpression was found in hepatocellular carcinoma (Hsu et al., 1999). The role of Gal-3 in viral hepatitis is not understood.

In order to explore possible role of Gal-3 in the development of MCMV-induced hepatitis we used Gal-3 deficient mice on C57BL/6 background and two strains of MCMV. Our results provide the first evidence that Lgals3 deletion promotes MCMV-induced liver inflammation and enhances MCMV-induced hepatitis by facilitation of TNF-α-dependent hepatocyte death. This effect appears to be independent of NK cells. Moreover, TNF-α blockade before MCMV infection attenuates apoptosis of hepatocytes. Treatment of MCMV-infected Gal-3 KO mice with recombinant Gal-3 reduces liver necrosis and inflammation. Thus, our data show that Gal-3 plays an important role in MCMV-induced liver damage and therefore may be a potential target for therapeutic intervention in acute CMV-induced liver disease.

## Materials and Methods

### Mice

Gal-3–deficient mice on the C57BL/6 background (Gal-3 KO) and wild-type (WT) C57BL/6 mice (6–8 weeks of age) were used in the experiments. Breeding pairs of WT and Gal-3 KO mice were obtained from the University of California, Davis, United States (Davis, CA, United States; by courtesy of D.K. Hsu and F.T. Liu). All animal procedures were approved by the Ethics Committee of Faculty of Medical Sciences, University of Kragujevac and conducted in accordance with the National Institutes of Health guidelines for humane treatment of laboratory animals. Unless otherwise stated each experimental group in each experiment contained six animals.

### Viruses

The bacterial artificial chromosome (BAC)-derived MCMV strain MW97.01 has previously been shown to be biologically equivalent to MCMV strain Smith (VR-1399) and is hereafter referred as WT MCMV (Wagner et al., 1999). Mice were injected intraperitoneally (i.p.) with 1 × 10^5^ PFU of MCMV strain MW97.01 in a volume of 200 μL of diluent (PBS). Mice were also infected with MW97.01, the mutant virus lacking m157 gene (Δm157 MCMV) intravenously (i.v.) with 2 × 10^5^ PFU in a volume of 100 μL of diluent (PBS).

### Serum Levels of Transaminases

Serum levels of asparate aminotransferase (AST) and alanine aminotransaminase (ALT) were measured 36 and 72 h after MCMV infection by standard photometric method using the automated biochemistry analyzer Olympus AU 400 (Olympus Diagnostica GMBH, Hamburg, Germany) and Olympus AU reagents, according to the manufacturer’s instructions, expressed in U/L.

### Histological Analyses

The isolated livers were fixed in 10% phosphate-buffered formalin, embedded in paraffin, and consecutive 4 μm tissue sections were cut at various depths and mounted on slides. Sections were stained with Hematoxylin and Eosin (H&E) and every fourth (six slides) was evaluated for inflammation and necrosis in the liver. Section examined under low-power light microscopy (BX51; Olympus) equipped with digital camera. Scores of cumulative liver pathology for inflammation and necrosis were presented using the following scoring system: 0, normal (no pathology); 1, mild (1–3 abnormal areas); 2, moderate (3–5 abnormal areas); 3, severe (>5 abnormal areas). To determine the number of inflammatory infiltrates, whole liver tissue was sectioned at three non-subsequent depths and ten different fields were counted/section. Histological samples were blinded prior to evaluation.

### Immunohistochemical Detection of Galectin-3 and TNF-α

Formalin-fixed, paraffin-embedded mouse liver tissue sections were incubated with rabbit anti-TNF-α (ab66579, Abcam), rabbit anti-caspase-3, active/cleaved (NB100-56113, Novus Biologicals), anti-caspase 3 and rabbit anti-Gal-3 (ab53082, Abcam). Sections were visualized by rabbit-specific conjugate (Expose Mouse and RabbitSpecific HRP/DAB Detection IHC Kit; Abcam) and photomicrographed with a digital camera mounted on light microscope (BX51; Olympus). Virus-infected cells were visualized by anti-IE1 staining (MCMV protein expressed with early kinetics).

### Detection of Cell Death by TUNEL Staining

TUNEL (terminal deoxynucleotidyl transferase mediated dUTP nickend labeling) staining was performed to assess death hepatocytes in livers sections. Formalin-fixed, paraffin-embedded tissue sections were stained with *in situ* Cell Death Detection Kit, POD (Roche) following the instructions of manufacturer. DAB (3,3′-diaminobenzidine) as peroxidase substrate, was used to yield the characteristic brown color for nuclei. Slides were counterstained with hematoxylin solution and photomicrographed with a digital camera mounted on light microscope. The TUNEL-positive nuclei (brown) were quantified under ×400 magnification in five randomly fields and the data were summarized as the mean number of positive cells.

### Isolation of Hepatic Mononuclear Cells and Flow Cytometry

The isolation of liver-infiltrating inflammatory mononuclear cells was conducted as previously described (Volarevic et al., 2012). The isolated liver-infiltrating mononuclear cells were stained with fluorochrome-conjugated antibodies, including CD3, CD49b, CD8, NKG2D, CD69, perforin, granzyme B, NF-κB, IFN-γ, IL-10, IL-17, and TNF-α. Isotype Abs with matching conjugates were used as negative controls. For intracellular staining, cells were activated with PMA/ionomycin and processed as previously described (Milovanovic et al., 2012). Cells were analyzed with the FACSCalibur Flow Cytometer (BD Biosciences), and analysis was conducted with FlowJo (Tree Star).

### Infliximab Treatment

In order to inhibit production of TNF-α, mice were injected with chimeric monoclonal antibody, Infliximab (Remicade, JANSSEN BIOLOGICS B.V.), 5 mg/kg in 200 μL of saline intraperitoneally 1 h before MCMV infection. Mice were sacrificed 48 h after infection.

### Treatment With Recombinant Galectin-3

WT and galectin-3 KO mice were treated with recombinant Galectin-3, 5 μg per mouse (Peprotech, Rocky Hill, NJ, United States) intraperitoneally, 2 h before MCMV infection. Mice were sacrificed 36 h after MCMV infection.

### Isolation of Hepatocytes and Flow Cytometry

Hepatocytes were isolated as previously described (Li et al., 2010). Briefly, extirpated livers were transferred HBSS, cutinto 1 mm^3^ size pieces and washed in complete DMEM. Dissected tissue was centrifuged at 800 × G for 4 min, pellet resuspended in digestion medium (0.6% NaCl, 0.05% KCl, 1.2% HEPES, 0.07% CaCl2, 3 g/mL collagenase type I) and incubated for 20 min at 37°C. After incubation cells centrifuged at 800 × G for 4 min, pellet was washed twice in a complete DMEM, passed through the 100 μm filter and cells centrifuged at 600 × G for 4 min. Pellet that contains hepatocytes was resuspended in DMEM medium with FBS. Isolated hepatocytes were washed in cold PBS and resuspended in 1X binding buffer (10X binding buffer: 0.1 M Hepes/NaOH (pH 7.4), 1.4 M NaCl, 25 mM CaCl2) at concentration 1 × 10^6^/mL. Annexin FITC and propidium iodide (PI) were added to the 100 μL of cell suspension and incubated for 15 min at room temperature (25°C) in the dark. After incubation 400 μL of 1X binding buffer was added to each tube and stained cells were analyzed within 1h using FACSCalibur (BD, San Jose, United States) and FlowJo software (Tri Star). For detection of cell surface expression of calreticulin, isolated hepatocytes were stained with anti-calreticulin antibody (Abcam) and analyzed by FACSCalibur (BD, San Jose, United States) and FlowJo software (Tri Star).

### Measurement of TNF-α and HMGB1

Levels of TNF-α and HMGB1 in the liver homogenate were measured using ELISA kits (R&D Systems, Minneapolis, MN, United States for TNF-α and Elabscience for HMGB1) according to the manufacturer’s instructions.

### Statistical Analysis

All statistics were carried out using SPSS 18.0 for Windows software. Results were analyzed using the Student’s *t*-test or Mann–Whitney test and ANOVA or Kruskal–Wallis. Data in this study were expressed as the mean + SE or +SD. Values of *P* < 0.05 were considered significant.

## Results

### MCMV Infection Increases the Expression of Galectin-3 in Hepatocytes

**FIGURE 1:**
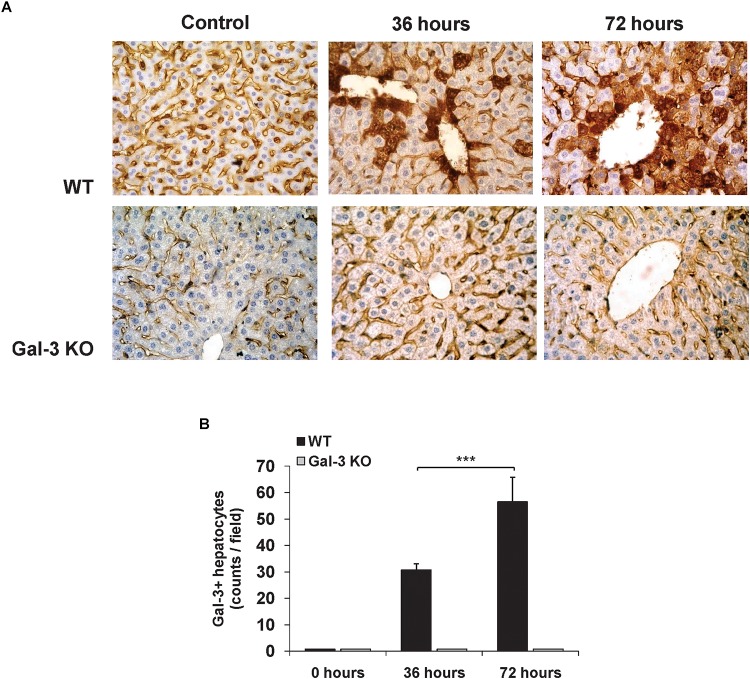
Acute MCMV infection increases Gal-3 expression in hepatocytes. **(A)** Representative sections of Gal-3 expression in the liver of WT and KO mice 36 and 72 h after MCMV infection with 1 × 10^5^ PFU/animal and the control uninfected liver from WT and KO mice. **(B)** Liver tissue was sectioned at three non-subsequent depths. Gal-3 positive hepatocytes were counted in ten different fields and presented as mean number + SD of positive cells per section, six mice per group, ^∗∗∗^*p* < 0.001.

Previously, we have shown very weak expression of Gal-3 in the liver parenchyma and biliary epithelial cells in healthy C57BL/6 mice (Arsenijevic et al., 2016). Also we found strongly enhanced expression of Gal-3 in patients with virus induced hepatitis (Volarevic et al., 2015). To explore the effect of MCMV infection on Gal-3 expression in mouse livers, immunostaining of Gal-3 in the livers of WT and Gal-3 KO mice was done 36 and 72 h after MCMV infection. Time-dependent increase of Gal-3 expression in hepatocytes after MCMV infection was noticed in the livers of WT mice (Figure 1A). Significantly higher number of Gal-3 expressing hepatocytes per field was noticed 72 h after infection when compared with liver sections obtained 36 h after MCMV infection (Figure 1B). As a control, Gal-3 was not detected in the livers of untreated and KO infected mice (Figure 1A).

### The Abscence of Gal-3 Enhances Virus Induced Hepatitis and MCMV Titers in the Liver

**FIGURE 2:**
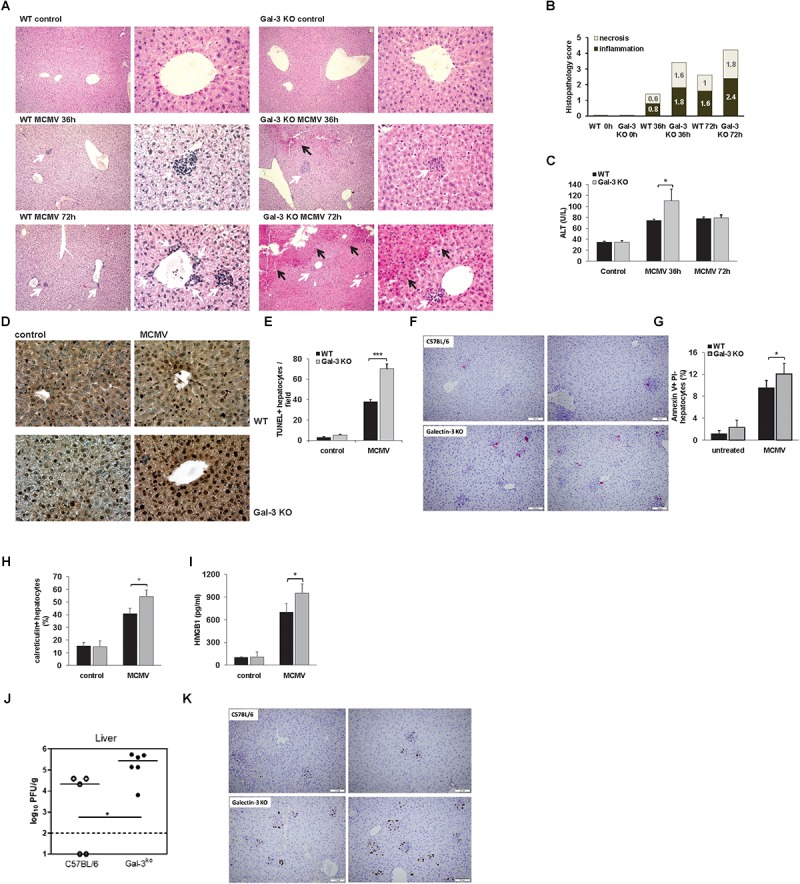
Galectin-3 deficiency enhances MCMV-induced hepatitis. WT and Gal-3 KO mice were i.p. infected with 1 × 10^5^ PFU/animal. Livers were analyzed 36 and 72 h after virus infection. **(A)** H&E staining of paraffin embedded liver sections. Black arrows highlight necrotic areas, and white arrows indicate inflammatory foci. **(B)** Scores of cumulative liver pathology for apoptosis/necroptosis and inflammation; the height of each bar represents the mean of the total histological score (6 animals per group). **(C)** ALT levels were determined in the serum 36 and 72 h after virus infection. **(D)** TUNEL staining of liver sections 72 h after MCMV infection. **(E)** Quantitative analysis of cell death rate: TUNEL-positive nuclei (brown) were counted in five random fields, and the data were summarized as the mean number of positive cells. **(F)** Apoptotic cells were visualized by anti-caspase-3 staining (shown in red). Two representative images are shown/group. **(G)** Apoptosis of hepatocytes isolated from MCMV infected WT and KO mice was analyzed by flow cytometry using Annexin V (FITC) and PI double staining. **(H)** Concentration of HMGB1 in the liver tissue homogenate 36 h p.i. determinated by ELISA. **(I)** Percentage of hepatocytes expressing membrane surface calreticulin isolated from the livers 36 h p.i. determined by flow cytometry. **(J)** Viral titres in liver is determined by standard plaque assay at 3 days p.i. **(K)** Virus-infected cells are revealed by anti-IE1 staining (shown in brown). Two representative images are shown/group. The data are presented as means +SE, or means+SD, 7 mice per group, ^∗∗∗^*p* < 0.001; ^∗^*p* < 0.05; two tailed, unpaired Student’s *t*-test.

In light of increased Gal-3 expression in hepatocytes of MCVM-infected mice, we wanted to explore the role of Gal-3 in overall severity of MCMV-induced hepatitis. For this, histological and serological parameters of liver damage were analyzed in WT and Gal-3 KO mice, 36 and 72 h after MCMV infection. Histological parameters related to MCMV-induced hepatitis, liver inflammation and necrosis were more pronounced in Gal-3 KO mice, 36 and 72 h after infection (Figure 2A,B). No difference in the architecture of liver tissue was noticed between WT and Gal-3 KO uninfected mice (Figure 2A). Bigger necrotic areas and inflammatory foci were observed in the livers of Gal-3 KO mice in comparison with WT mice, 36 h after infection (Figure 2A,B). Similar differences between Gal-3 KO and WT mice in the size of necrotic areas were observed 72 h after infection also (Figure 2A,B). At this time point, there was no difference in the size of inflammatory foci between the two groups (Figure 2A), but Gal-3 KO mice had higher number of smaller inflammatory foci compared with WT mice (Figure 2A). Although HCMV infection in immunocompetent hosts is subclinical, it is often accompanied with elevated serum levels of transaminases. Thus, we examined the level of alanine transaminase (ALT) in the sera of infected mice. In line with histological findings, we observed significantly higher level of ALT in the sera of Gal-3 KO mice 36 h after MCMV infection, compared to WT mice (Figure 2C).

In order to further analyze the liver damage in WT and KO mice after MCMV infection we used TUNEL assay. As shown in Figure 2D,E, 72 h after MCMV infection, the livers of Gal-3 KO mice contains significantly higher number of TUNEL positive (brown nuclei) hepatocytes than the livers of WT mice. Moreover, higher number of apoptotic (caspase 3 positive) cells per one inflammatory infiltrate was detected in Gal-3 KO compared to the WT livers (Figure 2F, red dots). To confirm the increased apoptosis of hepatocytes in Gal-3 KO mice, we isolated hepatocytes from WT and Gal-3 KO mice 72 h after MCMV infection and measured the percentage of apoptotic cells by flow cytometry. In line with histological observations, we detected significantly higher percentage of apoptotic (Annexin V positive) hepatocytes isolated from infected Gal-3 KO mice, compared with those from WT mice (Figure 2G). Given the marked necrotic fields in the liver sections from Gal-3 KO mice (Figure 2A,B) and the ability of MCMV to induce necroptosis (Upton et al., 2017), we examined markers of necroptotic death, HMGB1 in liver tissue homogenates and membrane expression of calreticulin on hepatocytes, 36 h after infection. We have found significantly higher concentration of HMGB1 in the liver homogenates (Figure 2H) and higher percentage of hepatocytes expressing calreticulin on membrane surface (Figure 2I) in Gal-3 KO mice in comparison with infected WT mice.

In the livers of Gal-3 KO mice, there is a significant increase in viral titers compared to the livers of wild-type mice (Figure 2J). There was no significant difference in viral titers in lung and spleen between WT and Gal-3 KO mice, 72 h after infection (Supplementary Figure S1A). At 8 days post-infection (Supplementary Figure S1B), viral plaques in spleen were not detected with the exception of a very low titer in one Gal-3 KO animal. In lungs, liver and salivary gland, viral plaques were readily detected but no significant differences were observed between the two groups. Lastly, to demonstrate the presence of virus-infected cells, sections of liver were stained with an antibody against IE1, a MCMV protein expressed with early kinetics. As shown in Figure 2K, in the liver tissue of Gal-3 KO mice, higher number of infected cells was observed compared to the WT animals.

Together, previous data indicate protective role of Gal-3 in MCMV-induced hepatitis, possibly relating to its increased expression in hepatocytes and known role of Gal-3 in attenuation of cell death (Takenaka et al., 2004).

### Enhanced Disease in Gal-3 KO Mice Is Independent of NK Cell Activation

**FIGURE 3:**
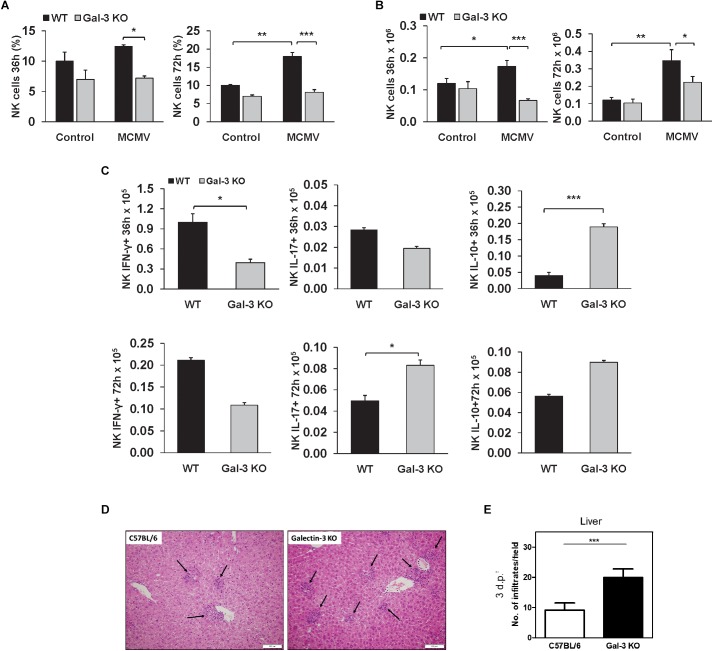
Liver inflammatory NK cells are attenuated in Gal-3 KO mice. Flow cytometry of mononuclear cells isolated from livers of WT and KO 36 and 72 h after i.p. infection with 1 × 10^5^ PFU/animal MCMV was done. Percentages **(A)** and absolute numbers **(B)** of NK cells calculated per liver. **(C)** Total numbers of IFN-γ+, IL-17+, and IL-10+ NK cells 36 and 72 h after infection calculated per liver. **(D)** Images of H&E stained liver sections obtained from C57BL/6 and KO mice 72 h after intravenous infection with MCMVΔm157 (2 × 10^5^ PFU/animal). **(E)** Number of inflammatory cell infiltrates (indicated by black arrows in **D**) determined by manual counting. Data are presented as the mean + SE, 15 mice per group, ^∗∗∗^*p* < 0.001; ^∗∗^*p* < 0.005; ^∗^*p* < 0.05; two tailed, unpaired Student’s *t*-test.

Taking into account the facts that NK cells play a crucial role in the early immune response against MCMV in C57BL/6 mice (Scalzo and Yokoyama, 2008) and that NK cells contribute to liver damage in viral infections (Zheng et al., 2015), we explored the possibility that bigger liver damage in infected Gal-3 KO mice is a consequence of stronger NK cell activity. We analyzed the presence and the phenotype of NK cells in liver mononuclear infiltrates in WT and Gal-3 KO mice, 36 and 72 h after MCMV infection. There was no statistically significant increase in the percentage and total number of NK cells in the livers of infected Gal-3 KO mice in comparison with uninfected Gal-3 KO mice (Figure 3A,B). On the other hand, MCMV infection induced a significant increase in both total number and percentage of NK cells in the livers of WT mice (Figure 3A,B). Total number of IFN-γ expressing NK cells 36 h after MCMV infection was significantly higher in the livers of WT mice in comparison with Gal-3KO mice, while 72 h after infection this difference lost significance (Figure 3C). No significant difference in the percentage and total number of NK cells expressing IL-17 was noticed between the groups. Lastly, total number of IL-10 positive NK cells was significantly higher in the liver of Gal-3 KO mice in comparison with WT mice, 36 h after infection (Figure 3C).

In order to exclude the role of NK cells in the higher liver damage in Gal-3 KO mice, we analyzed liver damage in WT and KO mice after infection with Δm157 MCMV, the mutant virus lacking m157 gene, which does not stimulate NK cells. As observed for WT MCMV, the inflammatory cell infiltrates were readily observed in the liver tissue of both wild-type and Gal-3 KO infected mice (Figure 3D, black arrows). No difference in the size of infiltrates was observed between the two groups but, interestingly, the number of infiltrates was significantly higher in Gal-3 KO mice compared to the wild-type mice (Figure 3E). Thus, enhanced liver damage in MCMV-infected Gal-3 KO mice does not appear to relate to NK cell activity.

### The Expression of TNF-α in Hepatocytes Is Increased in MCMV-Infected Gal-3 KO Mice

**FIGURE 4:**
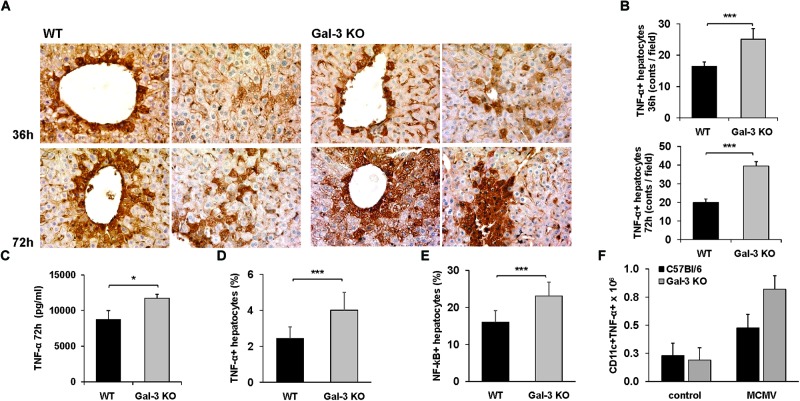
Galectin-3 deficiency enhances the expression of TNF-α in hepatocytes of infected animals. **(A)** Sections of TNF-α liver immunohistochemistry 36 and 72 h after MCMV infection. **(B)** Quantitative analysis of TNF-α positive hepatocytes: positive hepatocytes were counted in five random fields, and the data were summarized as the mean number of positive cells. **(C)** Concentration of TNF-α in the liver tissue homogenate determinated 72 h p.i. by ELISA. Percentage of TNF-α+ **(D)** and NF-κB+ **(E)** hepatocytes and **(F)** total number of CD11c+ TNF-α+ cells determined by flow cytometry 72 h after infection. Data are presented as the mean + SE, six mice per group, ^∗^*p* < 0.05, ^∗∗∗^*p* < 0.001, two tailed, unpaired Student’s *t*-test.

Based on our finding of enhanced necoptosis in Gal-3 KO mice and the fact that TNF-α signaling triggers necroptosis (Vandenabeele et al., 2010) and is required for MCMV-induced liver damage (Orange et al., 1997), TNF-α detection in the livers of MCMV-infected mice was done. The expression of TNF-α in hepatocytes was detected by immunostaing the livers of MCMV-infected mice both, WT and Gal-3 KO (Figure 4A). The number of TNF-α positive hepatocytes was significantly higher in the livers of Gal-3 KO mice compared to the group of infected WT mice, 36 and 72 h after infection (Figure 4B). Also, the concentration of TNF-α in the liver tissue homogenate was significantly higher in the group of Gal-3 KO mice compared to the group of WT mice, 72 h after MCMV infection (Figure 4C). Further, significantly higher percentage of TNF-α+ hepatocytes (Figure 4D), and total number of TNF-α+ CD11c+ cells (Figure 4E), analyzed by flow cytometry 72 h after MCMV infection, were found in the group of Gal-3 KO mice in comparison with WT mice. In accordance with the role of NF-κB in the promotion of TNF-α expression and its role in TNF-α mediated actions, significantly higher percentage of NF-κB+ hepatocytes was found in the group of Gal-3 KO mice in comparison to WT mice, 72 h after infection (Figure 4F). These results suggest that higher TNF-α expression in hepatocytes and immune cells could be the cause of enhanced MCMV-induced hepatocyte death observed in Gal-3 KO mice.

### Inhibition of TNF-α Binding Alleviates MCMV-Induced Hepatitis

**FIGURE 5:**
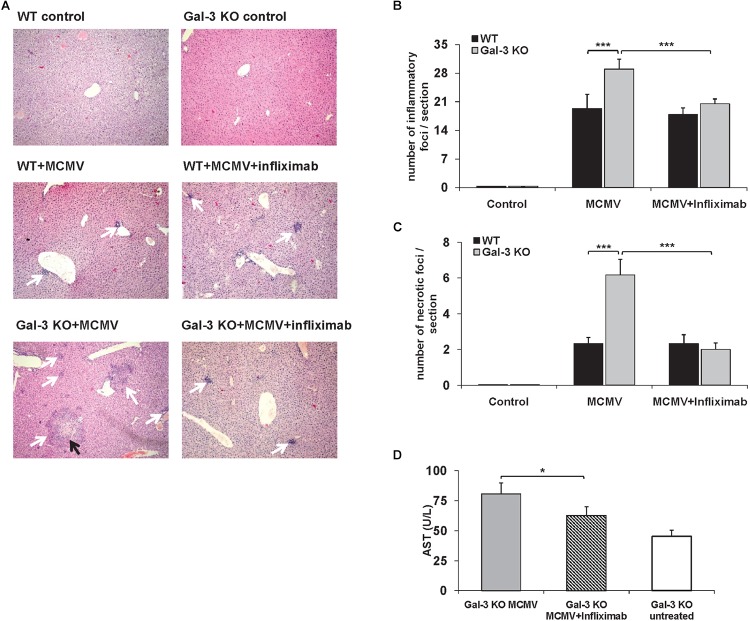
TNF-α blockade attenuates liver inflammation and necrosis in MCMV-infected Gal-3 KO mice. WT and KO mice were i.p. infected with 1 × 10^5^ PFU/animal. Chimeric monoclonal antibody, Infliximab (5 mg/kg) was administrated intraperitoneally 1 h before MCMV infection. Mice were analyzed 48 h p.i. **(A)** H&E staining of paraffin embedded liver sections. Black arrows highlight necrotic areas, and white arrows indicate inflammatory foci. **(B)** Number of inflammatory cell infiltrates per section (indicated by white arrows in **A**) was determined by manual counting. **(C)** Number of necrotic foci per section (indicated by black arrows in **A**) was determined by manual counting. **(D)** ALT levels determined in the serum. Data are presented as mean + SE of *n* = 6 mice. ^∗∗∗^*p* < 0.001; ^∗^*p* < 0.05 from two tailed, unpaired Student’s *t*-test.

In order to further explore the possibility that TNF-α plays a role in enhanced MCMV-induced liver damage in Gal-3 deficient animals, pharmacological inhibition of TNF-α with infliximab was done before infection. In the control group of WT mice, TNF-α blockade before MCMV infection did not alter liver inflammation or necrosis, while a pre-treatment of MCMV-infected Gal-3 KO mice with infliximab significantly decreased liver inflammation and necrosis (Figure 5A–C). Liver of untreated MCMV-infected Gal-3 KO mice contained bigger inflammatory and necrotic foci in comparison to the liver of infliximab-pretreated mice (Figure 5A). Also, the TNF-α blockade before MCMV infection significantly decreased serum levels of ALT in Gal-3 KO mice (Figure 5D). Ameliorated MCMV-induced hepatitis in Gal-3 KO mice treated with infliximab supports the role of TNF-α in enhanced disease in Gal-3 deficient animals.

### Exogenous Gal-3 Alleviates MCMV-Induced Liver Damage

**FIGURE 6:**
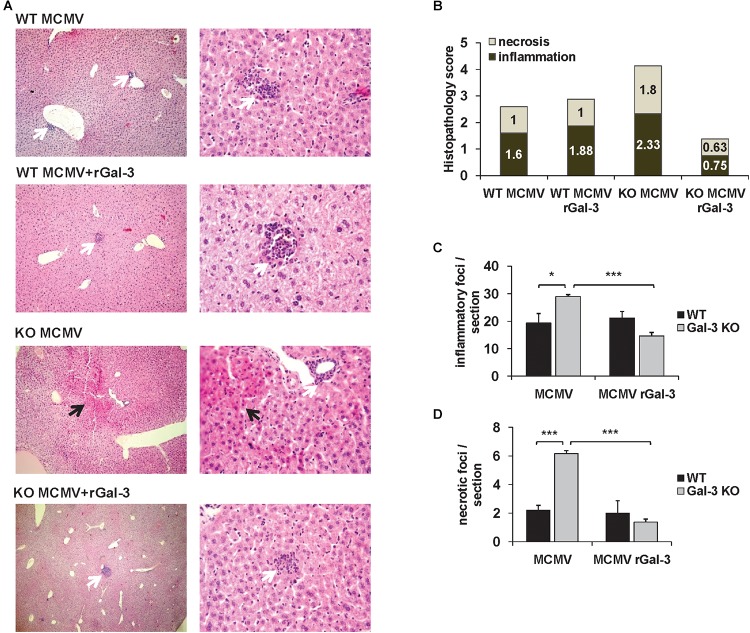
Gal-3 is protective in MCMV-induced hepatocyte damage. **(A)** H&E staining of paraffin embedded liver sections in WT and KO mice treated with recombinant Galectin-3 (50 μg/mL) intraperitoneally 2 h before MCMV infection, analyzed 72 h after infection. **(B)** Scores of cumulative liver pathology for apoptosis/necroptosis and inflammation. **(C,D)** Number of inflammatory cell infiltrates and necrotic areas per section determined by manual counting. Data are presented as mean + SE of *n* = 8 mice. ^∗∗∗^*p* < 0.001; ^∗^*p* < 0.05 from two tailed, unpaired Student’s *t*-test.

To confirm the protective role of Gal-3 in MCMV-induced hepatitis disease severity in WT and Gal-3 KO mice treated with recombinant Gal-3 was evaluated. Administration of recombinant Galectin-3 did not significantly alter inflammation and necrosis in WT mice (Figure 6A,B). Although we have not observed an altered number of inflammatory foci per liver in WT mice, there was an increase in their size (Figure 6A, white arrows). Gal-3 KO mice treated with recombinant Galectin-3 had lower histological score of necrosis and inflammation and lower number of inflammatory and necrotic foci per section, 72 h after MCMV infection (Figure 6A–D). Alleviated disease in Gal-3 KO mice treated with recombinant Galectin-3 confirms the protective role of Gal-3 in MCMV induced hepatitis.

## Discussion

Here, we provide the first evidence that Gal-3 plays a protective role in MCMV-induced hepatitis as indicated by higher liver damage, serum levels of ALT and higher virus titers in Gal-3 deficient mice (Figure 2). Further exogenous Gal-3 alleviates MCMV-induced liver damage (Figure 6).

Several clinical studies indicate an increased serum level of Gal-3 in viral infections (Ten Oever et al., 2013; Lukic et al., 2017), and point out Gal-3 as a potential marker of viral infection (Kobayashi et al., 2015). Findings obtained in animal model of coxsackievirus B3 virus induced cardiac injury in C57BL/6 mice studies indicate a protective role of Gal-3 in viral-induced diseases (Jaquenod De Giusti et al., 2015). Here, we show that Gal-3 gene deletion leads to accelerated liver damage induced by MCMV infection. In C57BL/6 mice, Gal-3 deletion enhances liver disease which is characterized by higher liver infiltrates and necrosis (Figure 2A,B), increased serum level of ALT (Figure 2C), enhanced apoptosis and necroptosis of hepatocytes (Figure 2D–I), higher viral titers (Figure 2J) and higher number of MCMV-infected cells in liver (Figure 2K). Further, a pretreatment of MCMV-infected Gal-3 KO mice with recombinant Gal-3 reduced the inflammation and liver damage in MCMV-induced hepatitis (Figure 6). The diverse effects of Gal-3 in different inflammatory conditions depend on the dominant pathogenic mechanisms involved. In macrophage mediated carditis and cardiac fibrosis induced by coxsackievirus B3 virus disruption of Gal-3 gene, or pharmacological inhibition of Gal-3, has the protective role (Jaquenod De Giusti et al., 2015). We also previously reported that Gal-3 gene deletion alleviates T and NKT cell mediated hepatitis (Volarevic et al., 2012, 2015). But, in line with finding of this study is our previous report that Gal-3 gene deletion aggravates xenobiotic induced PBC (Arsenijevic et al., 2016) due to enhanced hepatocyte apoptosis and release of autoantigens.

NK cells play a crucial role in the early immune response against murine cytomegalovirus infection and in the clearance of MCMV in C57BL/6 mice (Scalzo and Yokoyama, 2008). NK cells, also contribute to liver damage in viral infections (Zheng et al., 2015). Although NK cells express Gal-3 that mainly downregulates their inflammatory and cytotoxic activities (Radosavljevic et al., 2011), higher liver damage in Gal-3 KO mice was not accompanied with higher activity of NK cells (Figure 3). Here, we have demonstrated that WT mice had higher influx of NK cells in the liver in comparison with Gal-3 KO mice (Figure 3A,B). Higher total number of IFN-γ expressing and lower total number of IL-10 expressing NK cells was noticed in the livers of WT mice (Figure 3C). In accordance with previous report that NK cells as the main source of IFN-γ, which plays a pivotal role in the antiviral response (Jost and Altfeld, 2013), our results indicate that lower viral titers in the livers of WT mice (Figure 2J) are the result of enhanced activity of NK cells in these mice in comparison with Gal-3 KO mice. The absence of NK cells involvement in higher liver damage in Gal-3 KO mice was confirmed by experiments with MCMVΔm157 infection. The stimulation of activating Ly49H receptor of NK cells with MCMV-encoded protein m157, has a crucial role in early MCMV control and resistance to MCMV infection (Smith et al., 2002). The deletion of m157 gene or blocking the Ly49H receptor on NK cells abrogates the control of MCMV infection in most of the organs (Brown et al., 2001; Lee et al., 2001; Bubic et al., 2004; Sumaria et al., 2009). Higher number of liver infiltratesin Gal-3 KO mice infected with MCMVΔm157 in comparison with MCMVΔm157 infected WT mice (Figure 3D,E) suggests that higher activity of NK cells doesn’t play important role in enhanced liver damage of Gal-3 KO mice.

Acute MCMV infection generates an early systemic inflammatory response characterized by high levels of TNF-α, IL-12, IFN-γ, and type I IFNs (Orange and Biron, 1996; Nguyen et al., 2002). TNF-α mediates MCMV-induced liver damage independently of T and NK cells. It is required for the development of early hepatic necrotic foci and increased levels of liver enzymes (Orange et al., 1997). Our results indicate that hepatocytes are a significant source of TNF-α in MCMV infection (Figure 4). Although TNF-α is traditionally described as cytokine secreted by the cells of innate immunity (Wajant et al., 2003), tissue-specific cells also produce TNF-α (Gonzalez-Amaro et al., 1994; Bluml et al., 2012; Yoshigai et al., 2014). Production of TNF-α in the livers of MCMV infected mice depends on TLR3, TLR7, and TLR9 signaling (Zucchini et al., 2008; Crane et al., 2012) and also on activity of MAPK-activated protein kinase 2 (Ehlting et al., 2016). Since, we found MCMV-infected hepatocytes (Figure 2K) it can be assumed that MCMV directly stimulates TNF-α production in hepatocytes. We detected higher expression of TNF-α and higher number of TNF-α expressing hepatocytes in the liver of MCMV-infected KO mice (Figure 4). Additionally, TNF-α blockade with monoclonal antibody, Infliximab, significantly reduced hepatocyte death in MCMV-infected mice, especially in Gal-3 KO mice (Figure 5). TNF-α sensitizes hepatocytes to both caspase-dependant and caspase-independent apoptosis (Jones et al., 2000), and triggers necroptosis (Vandenabeele et al., 2010). Taking into account these observations, it appears that the higher production of TNF-α in hepatocytes of MCMV-infected Gal-3 KO mice contributes to greater liver damage.

Normal hepatocytes do not express Gal-3 in humans (Arsenijevic et al., 2016), but expression of Gal-3 is increased in different liver diseases (Shimonishi et al., 2001). Here, we have shown no Gal-3 expression in hepatocytes of untreated mice and a time-dependant increase of Gal-3 expression in hepatocytes of MCMV-infected WT mice (Figure 1A,B). This finding is in accordance with previously reported up-regulated expression of Gal-3 in mouse CNS tissue after encephalomyocarditis virus infection (Kobayashi et al., 2015) and in microglia and astrocytes of mice infected with Junin virus (Jaquenod De Giusti et al., 2011). Recently, it has been shown that TNF-α increases expression of Gal-3 (Okamoto et al., 2019), indicating that MCMV-induced Gal-3 expression in hepatocytes could be the result of virus-induced TNF-α production. The deletion of galectin-3-encoding gene sensitizes human keratinocytes, colorectal cancer cells, leukemia cells, human renal cell carcinoma and cholangyocarcinoma cells to apoptosis (Shi et al., 2007; Wongkham et al., 2009; Cheng et al., 2011; Xu et al., 2013), whereas the overexpression of Lgals3 protects the cells from apotosis (Takenaka et al., 2004). Gal-3 inhibits TNF-related apoptosis-inducing ligand, TRAIL, induced apoptosis by activation of the PI3K/Akt pathway which blocks loss of the mitochondrial membrane potential, resulting in inhibition of caspase-9 and caspase-3 activation (Oka et al., 2005). In accordance with this, we found higher percentage of TNF-α positive (Figure 4A–D) and apoptotic hepatocytes (Figure 2D–G) in infected Gal-3 KO mice, compared to WT mice. Further, we found significantly higher HMGB1 release (Figure 2H) and percentage of calreticulin positive hepatocytes (Figure 2I), markers of necroptotic death (Kepp et al., 2014), in the livers of infected Gal-3 KO mice compared with WT mice. HMGB1 binds to several receptors on the surface of immune cells (TLR2, TLR4, RAGE) and mediates strong inflammation (Scaffidi et al., 2002), while exposed calreticulin binds to receptors on antigen presenting cells and stimulates phagocytosis (Chao et al., 2010). It can be assumed that higher necroptosis of hepatocyte in Gal-3 deficient mice, early after MCMV infection, accompanied with higher release of HMGB1 (Figure 2J), enhances liver inflammation and TNF-α production in innate immune cells (Figure 4F) which in turn activates NF-kB in hepatocytes and subsequently augments the expression of innate cytokines (TNF-α) in hepatocytes (Zhou et al., 2016) leading to enhanced liver damage.

**FIGURE 7:**
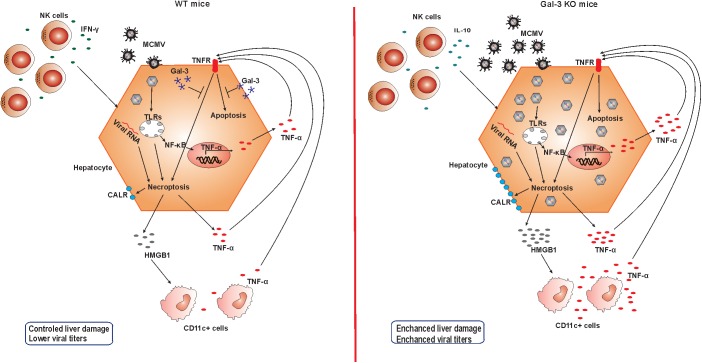
Role of Gal-3 in MCMV-induced hepatitis. In the presence of Gal-3 (left panel) liver infiltrating NK cells contain IFN-γ and take a role in viral clearance. MCMV in hepatocytes induce apoptosis and necroptosis, membrane expression of calreticulin (CALR), release of pro-inflammatory molecule HMGB-1, and TNF-α production. TNF-α binds TNFR and additionally stimulates apoptosis, but intracellular Gal-3 protects hepatocytes from cell death. Result is controlled liver damage and lower viral titers. In Gal-3 KO mice (right panel) NK cells that contain more IL-10 are poorer in controlling the virus, leading to increased virus-induced apoptosis and necroptosis. Increased death of Gal-3 KO hepatocytes is accompanied with higher expression of CALR and HMGB1 release that stimulates TNF-α in CD11c+ cells. More MCMV in Gal-3 KO hepatocytes induce higher NF-κB mediated production of TNF-α that augments apoptosis of hepatocytes, and in the absence of protective effect of Gal-3 results in more severe liver damage in Gal-3 KO mice.

Finally, our data suggest that the increased expression of Gal-3 in MCMV-infected livers protects hepatocytes from TNF-α facilitated apoptosis and necroptosis, and consequentially attenuates liver damage in MCMV-induced hepatitis (Figure 7). This findings combined with reduced MCMV-induced liver damage after recombinant Gal-3 treatment indicate protective role for Gal-3 in MCMV-induced liver damage, which can be of therapeutic relevance.

## Author Contributions

BS (1st Author), MM, NA, SJ, and ML conceived and designed the experiments. BS (1st Author), JM, AA, BS (4th Author), IS, and MM performed the experiments. BS (1st Author), IS, and MM analyzed the data. BS (1st Author), ML, and MM wrote the paper.

## Conflict of Interest Statement

The authors declare that the research was conducted in the absence of any commercial or financial relationships that could be construed as a potential conflict of interest.

## Supplementary Material

FIGURE S1 Galectin-3 KO mice exhibit higher viral titres early after infection. C57BL/6 and galectin-3 KO mice were i.v. infected with 2 × 10^5^ PFU/animal. Viral titres in individual organs are determined by standard plaque assay at **(A)** 3 days and **(B)** 8 days post-infection. Each dot represents an individual animal and the bar represents the median titre.
