# Supplementary figures and images for: Galectin-3 Deficiency Facilitates TNF-α-Dependent Hepatocyte Death and Liver Inflammation in MCMV Infection
Source: Front Microbiol. 2019 Feb 8;10:185. doi: 10.3389/fmicb.2019.00185 (PMC6376859; doi:10.3389/fmicb.2019.00185)

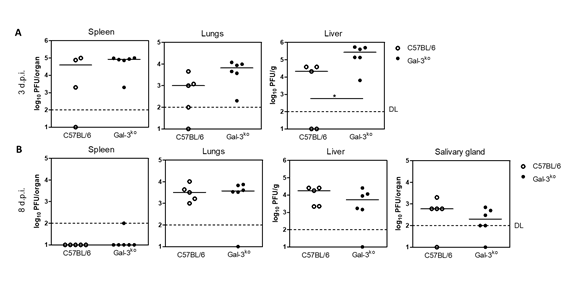

Supplement: FIGURE S1 — Galectin-3 KO mice exhibit higher viral titres early after infection. C57BL/6 and galectin-3 KO mice were i.v. infected with 2 × 10^5^ PFU/animal. Viral titres in individual organs are determined by standard plaque assay at (A) 3 days and (B) 8 days post-infection. Each dot represents an individual animal and the bar represents the median titre. [file Image_1.TIF]
